# Use of a Potential Probiotic, *Lactobacillus plantarum* L7, for the Preparation of a Rice-Based Fermented Beverage

**DOI:** 10.3389/fmicb.2018.00473

**Published:** 2018-03-14

**Authors:** Sib Sankar Giri, Shib Sankar Sen, Subrata Saha, Venkatachalam Sukumaran, Se Chang Park

**Affiliations:** ^1^Laboratory of Aquatic Biomedicine, College of Veterinary Medicine and Research Institute for Veterinary Science, Seoul National University, Seoul, South Korea; ^2^Molecular Parasitology Laboratory, School of Life Sciences, Jawaharlal Nehru University, New Delhi, India; ^3^Department of Mathematics, Institute of Engineering and Management, Kolkata, India; ^4^Department of Zoology, Kundavai Nachiyar Government Arts College for Women (Autonomous), Thanjavur, India

**Keywords:** fermented rice-beverage, *Lactobacillus plantarum* L7, probiotics, minerals, sugars, volatile compounds

## Abstract

This study aimed to isolate potential probiotic lactic acid bacteria from a traditional rice-based fermented beverage “bhaati jaanr” and to evaluate their role during preparation of the beverage. Among various isolates, *Lactobacillus plantarum* strain L7 exhibited satisfactory *in vitro* probiotic characteristics such as acid resistance and bile tolerance, cell surface hydrophobicity, auto-aggregation, antibiotic susceptibility, and antimicrobial activities. Therefore, performance of L7 as a starter culture in rice fermentation was determined during a 6-day rice fermentation study. *L. plantarum* L7 decreased the pH, associated with an increase in total titratable acidity and organic acid production up to the 4th day of fermentation. The highest concentrations of succinic acid (0.37 mg/g), lactic acid (4.95 mg/g), and acetic acid (0.36 mg/g) were recorded on the 3rd, 4th, and 5th days of fermentation, respectively. Saccharifying (148.13 μg/min g^−1^) and liquefying (89.47 μg/min g^−1^) activities were the highest on days 3 and 2, respectively, and thereafter, they decreased. Phytase activity and the cleavage of free minerals (sodium, calcium, magnesium, manganese, and ferrous) increased up to days 3–4. The concentration of various accumulated malto-oligosaccharides (glucose, fructose, maltotriose, and maltoterose) was noted to be the maximum on days 4 and 5. Furthermore, gas chromatography-mass spectrometry analysis indicated the presence of various volatile compounds. The fermented material also exhibited 1,1-diphenyl-2-picrylhydrazyl and 2,2′-azino-bis(3-ethylbenzothiazoline-6-sulphonic acid) radical scavenging activity. Therefore, the probiotic, *L. plantarum* L7, has a significant role in the fermentation of this beverage and enhances its functional properties.

## Introduction

For centuries, prior to the invention of pasteurization and sterilization, fermentation was used worldwide as a means of preserving food, and remains in use for this purpose even presently. Fermentation results in the breakdown of non-digestible carbohydrates; enriches the pool of essential amino acids, minerals, and vitamins; and enhances the overall quality, digestibility, and aroma of food (Ray et al., 2016). At present, more than 5,000 types of fermented foods are produced worldwide, many of which are traditional (Ray et al., 2016). In India, such foods are prepared from the most common cereals, including rice, corn, wheat, millet, and sorghum (Das et al., 2012), and their preparation remains a culinary skill. Cereals are the most important source of dietary proteins, carbohydrates, vitamins, minerals, and fiber for people worldwide. Cereals could be used to prepare cereal-based fermented beverages with probiotics if these formulations fulfill probiotic requirements and have acceptable physicochemical characteristics and organoleptic properties (Salmerón et al., 2015). Cereals have a potential to support the growth of probiotics that have been associated with the reduction of the risk of chronic diseases such as obesity, cardiovascular disease, and type II diabetes (Martins et al., 2013). Besides fulfilling the consumer demand for non-dairy beverages, cereal-based beverages can be potential sources of functional compounds such as antioxidants, dietary fiber, minerals, probiotics, and vitamins (Kreisz et al., 2008). Therefore, cereals have a great potential in the development of functional beverages that can promote gastrointestinal health and other beneficial properties.

Tamang et al. (2016) stated that fermented foods are the hub of consortia of microorganisms. Lactic acid bacteria (LAB) are often involved in the fermentation of food products to varying extents, having either positive or negative effects on the final product (Rhee et al., 2011). *Lactobacillus* species found in traditional fermented foods are suitable for food technology applications, as they can transform food into new products, are resistant to low pH, and exert antagonistic effects against harmful microorganisms (Rai et al., 2017). LAB and other starter cultures transform the biochemical and organoleptic characteristics of the substrates, produce various metabolites, and enrich foods with a range of micronutrients (vitamins, minerals, amino acids, etc.,), edible microbes that benefit health (i.e., probiotics), fermentable sugars (i.e., prebiotics), dietary fiber, phytochemicals, and digestive enzymes (Ray et al., 2016; Tamang et al., 2016). Furthermore, health improving properties of probiotic LAB are mostly associated with reducing symptoms of lactose intolerance, positive influence on the intestinal microbiota, improving intestinal function, growth inhibition of pathogenic microorganisms, production of B vitamins (especially folic acid), and stimulation of the immune response (Enujiugha and Badejo, 2017). Traditional alcoholic beverages vary from crystal-clear products and turbid liquids to thick gruels and pastes. In East Asia, the generic terms rice wine and rice beer are used in reference to alcoholic beverages made from rice, some well-known traditional examples of which include shaosingjiu (China), cheongju, maggolli (Korea), sake (Japan), and tapuy (the Philippines) (Rhee et al., 2011). In India, customary rice-based fermented beverages include haria, apong, jou, judima, zutho, bhaati jaanr, and rice jaan. These products are made using unique and traditional fermentation techniques. For example, bhaati jaanr, consumed as a staple nutritious beverage by the Gorkhas in Northeast India, is prepared from cooked (for 15 min in open cooker) and air-dried glutinous rice (Tamang, 2001). Marcha, a dry mixed starter culture is powdered, and sprinkled (~2%) over cooked rice, mixed, and maintained at room temperature for 1–2 days in a vessel or an earthen pot for saccharification. Then, the vessel is closed airtight and the food product is fermented for few days (Tamang and Thapa, 2006). Saccharification and fermention are driven by LAB, molds, and yeasts present in the starter culture. Similarly, haria is prepared using boiled, scorched rice, which is mixed with a starter culture named bakhar (1:100), transferred to an earthen pot, stored under dark conditions for 3–5 days for fermentation, and diluted with water. It is principally consumed by the ethnic groups of Central and East India (Ghosh et al., 2014). These alcoholic beverages have been found to have many ethno-medicinal properties (Ray et al., 2016) that have yet to receive scientific attention. However, lesser attention has been paid to the development of cereal-based fermented probiotic foods, despite cereals being the largest dietary component for the majority of the populations in developing countries. Further, during fermentation of cereal-based products, several volatile compounds are formed which contribute to a complex blend of flavors (Gupta and Abu-Ghannam, 2010). However, the analysis of volatile flavor compounds in cereal-based probiotic products has not yet been studied in detail.

Among the LAB commonly associated with food fermentation processes, members of the genera *Lactobacillus* and *Bifidobacterium* generally predominate. The association between LAB or *Saccharomyces* and the preparation of traditional rice-based fermented beverages has been reported previously (Tamang and Thapa, 2006; Ghosh et al., 2015a,b); however, the role of indigenous bacteria in their production has been only rarely studied. Further, isolation and characterization of new strains of lactobacilli from traditional fermented foods can present a dual advantage of revealing taxonomic characteristics and obtaining strains with interesting functional traits that may be useful for biotechnological and/or probiotic applications (Ortu et al., 2007). Therefore, in the present investigation, LAB with potential probiotic properties was isolated from a sample of the rice-based fermented beverage, bhaati jaanr. Further, screened potential probiotic LAB strains were used as a starter culture for the fermentation of rice and performance of the starter culture was monitored by microbial dynamics and metabolite evaluation during the fermentation. Further, physico-chemical changes, antioxidant activity, and mineral contents in the fermented beverage were investigated. Presence of various volatile compounds in the fermented product was also analyzed.

## Materials and methods

### Isolation of lactobacilli

Bhaati jaanr was collected directly from a household in a village in Darjeeling District, West Bengal, India, and transported to the laboratory under refrigerated conditions. One hundred microliters was pour plated into Rogosa SL agar (Sigma-Aldrich, St. Louis, MO, USA) supplemented with 1.32 mL/L acetic acid, and placed in an incubator containing 5% CO_2_ in air at 35°C for 48 h. Several colonies with an LAB-like morphology were picked at random, and their purity was verified by repeated streaking and sub-culturing on fresh Rogosa SL agar. Isolates were then Gram stained and tested for catalase production. In total, nine Gram-positive and catalase-negative isolates, designated L1–L9, were selected for further study. The screened LAB isolates were stored at −20°C in MRS broth containing 25% (w/v) glycerol.

### *In vitro* probiotic characteristics

#### Acid resistance and bile tolerance

Acid resistance was tested using MRS broth adjusted with HCl to a final pH of 2.0 and 3.0, and bile tolerance was assessed with MRS broth containing 0.3% (w/v) oxgall (Oxoid, Basingstoke, UK). Young broth cultures of the isolates were inoculated into these media at 10^7^ CFU/mL and incubated under anaerobic conditions (including 5% CO_2_) for 4 h. Survival rates were determined based on the number of viable cells present on MRS agar after spread plating.

#### Cell surface hydrophobicity

Isolate cell hydrophobicity was determined following the method of Ekmekci et al. (2009), which is based on the affinity of cells (cultured overnight in MRS broth) for toluene in a two-phase system. Hydrophobicity was calculated according to the percentage decrease in optical density at 600 nm (OD_600_) of the original bacterial suspension due to cells partitioning into the hydrocarbon layer, as follows:

Hydrophobicity (%) = 100 × ([OD_600_ before mixing − OD_600_ after mixing] / OD_600_ before mixing).

#### Auto-aggregation assay

The auto-aggregation ability of each isolate was measured according to the method described by Shin et al. (2012). Bacterial cells were collected by centrifugation (9,400 × *g* for 3 min), washed twice with PBS, resuspended in PBS, and vortexed for 30 s. OD_600_ was measured at 0 h and 4 h using a UV/visible spectrophotometer. Auto-aggregation ability was expressed as the auto-aggregation percentage (AA%), calculated as follows:

AA% = (1 − A_t_/A_0_) × 100,

where A_t_ = absorbance at 600 nm at 4 h and A_0_ = absorbance at 600 nm at 0 h.

#### Antibiotic susceptibility

The minimum inhibitory concentrations (MICs) of chloramphenicol, ampicillin, clindamycin, neomycin, erythromycin, gentamycin, kanamycin, vancomycin, and streptomycin with respect to the selected lactobacilli were determined using a broth microdilution test that has been described previously (Garcia et al., 2016). The MIC of each antibiotic was determined as the lowest concentration inhibiting visible bacterial growth. The cut-off values specified by the European Food Safety Authority (2012) were used to categorize the isolates as susceptible or resistant to each tested antibiotic.

#### Antagonistic activity against pathogens

The lactobacilli were cultured in MRS broth for 18 h at 35°C under anaerobic conditions (including 5% CO_2_) and centrifuged at 10,000 × *g* at 4°C for 15 min. Supernatants were then collected and neutralized (to pH 6.8) with 5 N NaOH. A 200-μl suspension of the indicator bacterium was then incorporated into a 20-mL MRS plate (for a final concentration of approximately 10^6^ CFU/mL). Wells 5 mm in diameter were created, into which, 100-μl aliquots of the lactobacillus supernatant were dispensed. The plates were incubated under aerobic conditions at 35°C for 48 h. An agar plate with wells containing MRS broth was used to determine the inhibitory activity of the medium (control). After the incubation period, antagonistic activity was recorded as the diameter (in mm) of the growth inhibition zones around the wells. Zones with a diameter greater than 1 mm were considered indicative of positive inhibitory activity (Garcia et al., 2016).

### Identification of isolate L7

L7, the isolate with the most promising probiotic qualities, was identified based on morphological, physiological, and biochemical characteristics, as well as 16S rRNA gene sequencing. Bacterial genomic DNA was extracted using a genomic DNA extraction kit (QIAGEN, Hilden, Germany) according to the manufacturer's instructions. PCR was carried out using primers 27F (5′-AGAGTTTGATCCTGGCTCAG-3′) and 1492R (5′-GGTTACCTTGTTACGACTT-3′) (Garcia et al., 2016), the products of which were purified with a QIAquik PCR Purification Kit (QIAGEN) and sequenced on an ABI Prism automatic DNA sequencer using a BigDye Terminator Cycle Sequencing Kit (Applied Biosystems, Foster City, CA, USA). The NCBI Basic Local Alignment Search Tool (BLAST; http://www.ncbi.nlm.nih.gov/) was used to compare sequences and calculate percentage similarity.

### Rice fermentation by isolate L7

Isolate L7 exhibited the most effective probiotic characteristics *in vitro* (as described in earlier section), and was therefore used in a fermentation test. One hundred grams of rice (*Oryza sativa* L.) was boiled (at a rice:water ratio of approximately 1:1.5), cooled to room temperature, and aseptically poured into a 1-l conical flask. A young culture of isolate L7 was inoculated (1% v/v; at a final concentration of 10^6^ CFU/mL) into the flask, thoroughly mixed, and incubated for 6 days under anaerobic conditions (including 5% CO_2_) at 35°C. The experiments were performed in three independent assays. Samples were collected every 24 h and stored at −20°C.

#### Enumeration of microorganisms

Sample (1 mL) was serially diluted with 0.1% (w/v) bacteriological peptone (Sigma). Diluted samples (0.1 mL) were spread plated (triplicate) onto MRS Agar plates. The plates were incubated under anaerobic conditions for 72 h at 37°C, and the viability was recorded as colony forming units (CFU) per mL, and cell concentration was expressed as log CFU/mL.

#### Measurement of pH and total titratable acidity (TTA)

The pH of each sample was tested with a digital pH meter (Sigma Instruments, USA). TTA was measured by homogenizing 10 g sample with 90 mL distilled water, followed by titration with 0.1 N NaOH using 0.1% (w/v) phenolphthalein in 95% ethanol as an indicator.

#### Enzyme activity assays

A 10-g sample was homogenized and centrifuged at 10,000 × *g* for 15 min, and the supernatant was collected and analyzed for α-amylase, glucoamylase, and phytase activity, with fresh supernatant being used for each test. α-Amylase activity was measured using starch solution as a substrate (Rai et al., 2017). Briefly, 250 μl supernatant was mixed with 250 μl 1% (w/v) starch solution and incubated for 40 min at 37°C. The reaction was terminated using 2 mL dinitrosalicylic acid (Sigma-Aldrich, USA) and boiling for 15 min. Absorbance at 550 nm was subsequently measured using a spectrophotometer. One unit of α-amylase activity was defined as the amount of enzyme needed to produce 1 μmol reducing sugars (glucose equivalent) per minute under assay conditions.

Glucoamylase activity was determined according to the method of Ghosh et al. (2015b), with slight modification. Briefly, 1 mL supernatant was mixed with an equal volume of 1% starch solution and incubated for 20 min at 37°C. The reaction was terminated by adding 12 N H_2_SO_4_, and the amount of glucose produced was measured using a glucose assay kit (Sigma-Aldrich, USA). One unit of glucoamylase activity was defined as the amount of enzyme needed to produce 1 μmol glucose equivalent per minute from starch under assay conditions.

Phytase activity was measured following the method of Shimizu (1992). Briefly, 150 μl supernatant was mixed with 600 μl substrate (0.2% [w/v] sodium phytate (Sigma-Aldrich, USA) in 0.1 M sodium acetate buffer, pH 5.0) and incubated for 30 min at 39°C. The reaction was stopped using 750 μl 5% trichloroacetic acid. Released inorganic phosphate was measured by adding 750 μl color reagent, which was prepared daily by mixing four volumes of 1.5% (w/v) ammonium molybdate in 5.5% (v/v) sulphuric acid solution and one volume of 2.7% (w/v) ferrous sulfate solution. Absorbance was then measured at 700 nm. One unit of phytase activity was defined as the amount of enzyme required to liberate 1 nmol phosphate per minute under assay conditions.

#### Quantification of sugars, organic acids, and alcohol

Quantification of sugars, acids, and alcohol was carried out following the methods described earlier (Miguel et al., 2012; Ghosh et al., 2015b). For this, a 10-mg sample was mixed with 30 mL 50 mM Tris-HCl (pH 8.8), incubated at 4°C for 1 h, and centrifuged at 20,000 × *g* for 20 min (Ghosh et al., 2015b). The resulting supernatant was tested after being passed through a filter with a pore size of 0.22 mm (Millipore, Billerica, MA, USA). Carbohydrate (glucose, maltose, fructose, maltotriose, and maltotetrose), organic acid (acetic acid, lactic acid, and succinic acid), and alcohol (ethanol) analyses were performed with an HPLC instrument (Agilent Technologies, New Delhi, India) equipped with a dual-detection system consisting of UV-vis (SPD 10Ai) and refractive index (RID-10Ai) detectors.

For sugars, a carbohydrate-NH_2_ column and a mobile phase comprising acetonitrile and water (3:1) were used. The temperature was maintained at 30°C and the flow rate was 1 mL/min. Malto-oligosaccharides (Sigma-Aldrich, USA), fructose, and glucose were used as standards. Organic acids and alcohol were detected by UV absorbance (210 nm) and RID measurements, respectively. An operating temperature of 30°C for ethanol, and 50°C for organic acids was used. Elution was carried out at 60°C with a flow rate of 0.6 mL/min, using 10 mM H_2_SO_4_ as eluent. Individual compounds were identified by comparing their retention times with that of standards. All the samples were analyzed in triplicate.

#### Determination of mineral content

A 5-g sample was homogenized in 25 mL deionized distilled water, centrifuged at 12,000 × *g* for 10 min, and the supernatant collected for analysis. The concentration of minerals in the supernatant was then determined by atomic absorption spectrophotometry (PerkinElmer) and flame photometry methods (Association of Official Analytical Chemists, 2005).

#### Measurement of 1,1-diphenyl-2-picrylhydrazyl (DPPH) and 2,2′-azino-bis(3-ethylbenzothiazoline-6-sulphonic acid) (ABTS) radical scavenging activity (RSA)

RSA was evaluated using DPPH and ABTS radicals, employing the modified method of Freire et al. (2017). Briefly, 3.9 mL DPPH solution (0.06 mM in methanol) was incubated with a 0.1-mL sample for 80 min in the dark, and absorbance at 515 nm was subsequently measured. For the blank control, 0.1 mL methanol was used in place of the sample. For the ABTS test, a 30-μl sample was incubated with 3 mL ABTS+^·^ radical for 6 min in the dark, and absorbance at 734 nm was then measured. As a blank control, 30 μl ethanol was used instead of the sample. RSA was calculated as follows:

RSA (%) = (1 − A_sample_ / A_control_) × 100,

where A_control_ is the absorbance of the blank, and A_sample_ is the absorbance of the sample.

#### GC-MS analysis

Fermented sample collected at 5th day was used for GC-MS analysis. Volatile compound profiles were determined according to the method described by Freire et al. (2015), with minor modifications. Sample was centrifuged twice (10,000 g for 10 min at 4°C), and 7 mL supernatant was put into 10 mL culture tube. And, extraction was done by stirring the sample with 700 μL of GC grade chloroform (Sigma-Aldrich, USA) for 15 min over agitation with a magnetic stirrer. After cooling at 4°C for 10 min, the organic phase was separated by centrifugation at 5,000 g for 5 min at 4°C. The extract was recovered into a vial using a Pasteur pipette. To this 2 volumes of BSTFA [bis (trimethyl silyl) trifluoroacetamide; Sigma-Aldrich, USA) were added. The tubes were capped tightly and were heated at 80°C in water bath for 1 hr. The samples were then dried with anhydrous sodium sulfate (Sigma-Aldrich, USA) and stored at −20°C. They were re-dissolved in 200 μl of hexane prior to GC/MS analysis.

Extracted sample was analyzed using a GC-MS system (Shimadzu QP2010 Plus, Japan) equipped with a capillary column (DB-5 MS; 0.25-mm film thickness, 0.25 mm i.d., 30 m length). Briefly, the carrier gas was helium at 49.5 kPa, which corresponds to a linear speed of 15.5 cm/s. The injection port temperature was 230°C. The column temperature was held at 50°C for 5 min and then programmed to rise from 50 to 200°C, at 3°C/min. Held at 200°C for 10 min and then programmed to go from 200 to 240°C, at 10°C/min and this temperature was held for 10 min. Electron impact spectra were acquired in positive ionization mode between m/z 45 and 500.

### Statistical analysis

The obtained data were analyzed by one-way ANOVA, and Tukey's test was employed to assess differences between the treatments. All statistical analyses were performed using OriginPro software (version 8; OriginLab Corporation, Northampton, MA, USA). *P* values < 0.05 were considered to indicate statistical significance, and results are expressed as means ± SD.

## Results and discussion

### *In vitro* probiotic characteristics of lab isolates

#### pH and bile tolerance

The pH of the gastric juice is considered among the vital factors affecting the survival of probiotic bacteria during their passage through the stomach to the intestine. Indeed, previous studies have shown that the viability of LAB between pH 2 and 4 is an important indicator of potential probiotic performance (Garcia et al., 2016). Interestingly, isolates L4 and L7 exhibited adequate levels of viability at pH 2.0 and 3.0 (Table 1). Tolerance of bile during transit through the gastrointestinal (GI) tract is essential for probiotic LAB to survive, grow, and exert their beneficial effects (Jena et al., 2013). In the present study, of the 9 isolates tested, L4 and L7 retained the highest levels of viability in 0.3% oxgall (Table 1). Survival of LAB in low pH and bile salts has been reported in several prior studies (Santos et al., 2016; Freire et al., 2017). Overall, isolate L7, followed by L4 appeared mostly to be able to survive and grow at low pH values and in the presence of 0.3% bile.

**Table 1:** Assessment of viability of isolated strains to low pH and bile salts during 4 h of incubation.

	**Final counts (Log CFU/ml) of isolated *Lactobacillus* strains**								
	**L1**	**L2**	**L3**	**L4**	**L5**	**L6**	**L7**	**L8**	**L9**
**pH**									
Control (0 h)	7.9 ± 0.11	7.7 ± 0.13	7.7 ± 0.10	8.0 ± 0.07	7.9 ± 0.16	7.8 ± 0.06	7.9 ± 0.11	8.1 ± 0.14	7.9 ± 0.11
pH 2.0 (4 h)	2.7 ± 0.05	1.9 ± 0.08	3.4 ± 0.12	5.2 ± 0.16	0	1.7 ± 0.12	5.8 ± 0.21	0	0.8 ± 0.06
pH 3.0 (4 h)	4.1 ± 0.24	3.5 ± 0.19	4.3 ± 0.14	6.3 ± 0.27	1.3 ± 0.11	3.2 ± 0.24	7.1 ± 0.14	0.6 ± 0.03	1.2 ± 0.08
**BILE SALT**									
Control (0 h)	7.8 ± 0.23	7.72 ± 0.16	7.91 ± 0.19	7.83 ± 0.21	8.06 ± 0.13	7.63 ± 0.14	7.74 ± 0.26	7.93 ± 0.18	7.68 ± 0.2
0.3% (4 h)	2.4 ± 0.07	1.9 ± 0.04	5.6 ± 0.32	6.4 ± 0.24	4.2 ± 0.15	3.8 ± 0.17	6.9 ± 0.3	2.8 ± 0.14	5.1 ± 0.18

*Results are shown as mean ± S.D (n = 3)*.

#### Cell surface hydrophobicity and auto-aggregation

The health-promoting effects of LAB depend on various mechanisms, including adherence to epithelial cells. This process involves various interactions and correlates with cell hydrophobicity and aggregation ability (Collado et al., 2008; Angmo et al., 2016). The LAB isolates in the present study differed widely with respect to hydrophobicity, with L7 exhibiting the highest value (61.4 ± 1.9%), followed by L4 (47.8 ± 2.4%) (Table 2). These results suggest that the complexity of the cell surface mosaic resulting from hydrophobic and hydrophilic appendages and other macromolecular components might lead to differences in hydrophobicity. Furthermore, all the isolates exhibited some degree of auto-aggregation, with the highest level being demonstrated by L4 (41.4 ± 0.93%), followed by L7 (39.4 ± 1.4%) (Table 2). Through aggregation, LAB may attain a mass large enough to adhere to host mucosal surfaces, and thus, exert their probiotic effects. *Lactobacillus plantarum* strains isolated from fermented food products (Angmo et al., 2016; Santos et al., 2016) exhibited a high degree of hydrophobicity with a strong capacity for auto-aggregation.

**Table 2:** Assessment of cell-surface hydrophobicity (%) and auto-aggregation (%) activities of lactobacilli isolates as measured after 4 h of incubation.

**Lactobaccilli isolates**	**Cell-surface hydrophobicity (%)**	**Auto-aggregation (%)**
L1	21.4 ± 0.17	5.1 ± 0.11
L2	34.1 ± 1.2	17.8 ± 0.73
L3	13.6 ± 0.07	14.3 ± 0.81
L4	47.8 ± 2.4	41.4 ± 0.93
L5	18.3 ± 0.63	22.4 ± 1.03
L6	42.4 ± 1.4	28.6 ± 1.1
L7	61.4 ± 1.9	39.4 ± 1.4
L8	16.4 ± 0.44	7.3 ± 0.26
L9	39.4 ± 0.57	14.9 ± 1.03

*Results are presented as mean ± SD (n = 3)*.

#### Antibiotic susceptibility

Bacteria intended to be used as probiotics should not carry transmissible antibiotic-resistance genes as that may lead to the development of new antibiotic-resistant pathogens (Saarela et al., 2000). According to the breakpoints recommended by the European Food Safety Authority (2012), all the LAB isolates were susceptible to the tested antibiotics (with the exception of vancomycin), which is consistent with that reported in earlier studies (Garcia et al., 2016; Manini et al., 2016; Santos et al., 2016). However, these isolates were intrinsically resistant to vancomycin, an inhibitor of cell wall synthesis. This is in agreement with previous studies that have demonstrated this trait in lactobacilli, pediococci, and *Leuconostoc* spp. (Jena et al., 2013; Manini et al., 2016). This resistance is considered a natural property derived from the presence of d-alanine:d-alanine ligase-related enzymes and is used to separate them from other Gram-positive bacteria (Klare et al., 2007).

#### Antagonistic activity against pathogenic bacteria

The antagonistic activity of the LAB isolates against selected pathogenic bacteria is presented in Table 3. Of the 9 isolates, 5 (L2, L3, L4, L7, and L8) inhibited the growth of all of the pathogens tested, although the nature of the inhibitory substance(s) produced by these bacteria remains unknown. In general, strain L7 exhibited the strongest antagonistic activity (inhibition zones >4 mm for *Staphylococcus aureus* MTCC 737 and *Listeria monocytogenes* MTCC 1143, and >5 mm for *Escherichia coli* MTCC 443 and *Bacillus cereus* MTCC 6629), followed by L4. Notably, the inhibitory activity observed cannot be attributed to the acidity of the culture, since supernatants were neutralized (pH 6.8) before use. Previous studies have demonstrated inhibition of the growth of diverse pathogens by LAB strains originating from various food sources (Manini et al., 2016; Santos et al., 2016). The production of metabolites by probiotic bacteria with antimicrobial properties may be beneficial for food preservation and the prevention of foodborne pathogen growth (Garcia et al., 2016).

**Table 3:** Antagonistic activity of culture supernatants of LAB strains against pathogenic bacteria.

**LAB isolates^†^**	**Indicator strains**			
	***E. coli* (MTCC 443)**	***S. aureus* (MTCC 737)**	***Listeria monocytogenes* (MTCC1143)**	***Bacillus cereus* (MTCC 6629)**
L1	+	−	++	−
L2	+++	+	+	++
L3	+	+	++	+
L4	+++	++	++++	+++
L5	−	+	++	+
L6	++	−		+
L7	++++	+++	+++	++++
L8	++	+	+	++
L9	−	+	−	++

† *: − < 1 mm inhibition zone, +: > 1 mm inhibition zone, ++ > 2 mm inhibition zone, +++ > 4 mm inhibition zone, ++++ > 5 mm inhibition zone*.

#### Identification of isolate L7

Isolate L7, which exhibited excellent probiotic characteristics *in vitro*, was identified based on its biochemical and morphological attributes, and phenotypic identification was confirmed by 16S rRNA gene sequence analysis. Using BLAST, high identity scores were noted when the obtained 16S rRNA sequence (1220 bp) was compared to *Lactobacillus* sequences. The 16S rRNA gene sequence of strain L7 demonstrated the highest similarity value (99%) with the corresponding gene sequence of several strains of *L. plantarum*. The 16S rRNA gene sequence of this strain (*L. plantarum* L7) has been submitted to GenBank under the accession number MF370940.

#### Physiochemical changes during fermentation

Changes in pH and TTA levels during rice fermentation are shown in Table 4. *L. plantarum* L7 had a positive effect on fermentation, as it lowered pH over time. The pH was the lowest on day 4 (3.54) and increased slightly thereafter. The opposite trend was observed in TTA levels, which increased to 0.86% from an initial value of 0.04%, and subsequently decreased. The highest concentrations of lactic acid (4.95 mg/g), acetic acid (0.36 mg/g), and succinic acid (0.37 mg/g) were recorded on the 4th, 5th, and 3rd days of fermentation, respectively (Table 5). The presence of lactic acid in fermented beverages is more desirable as it attributes to the mild sour taste (Stroehle et al., 2006). The observed decrease in pH over the course of fermentation may be related to the rapid production of lactic acid by *L. plantarum* L7, which would also have resulted in an unfavorable environment for certain spoilage bacteria. This lowered pH is very important, as it has been reported that food formulations with pH values around 3.5–4.5 contribute to decreased pH in the GI tract and enhance the stability, and therefore, contribute to the beneficial effects of the consumed probiotics (Kailasapathy and Chin, 2000). Production of organic acids by LAB on starchy substrates has been documented previously (Puerari et al., 2015; Salmerón et al., 2015). Thus, using rice as a substrate, *L. plantarum* L7 decreased pH by secreting various organic acids, with optimum organic acid production occurring within 4–5 days of fermentation.

**Table 4:** Changes in pH, total titrable acidity (TTA), alcohol (ethanol) content during fermentation.

**Fermentation day**	**pH**	**TTA (% lactic acid)**	**Ethanol (%; w/v)**
0	6.76 ± 0.09	0.04 ± 0.01	ND
1	6.45 ± 0.05	0.21 ± 0.02	ND
2	5.59 ± 0.03	0.58 ± 0.05	0.06 ± 0.01
3	4.68 ± 0.11	0.73 ± 0.02	0.14 ± 0.03
4	3.54 ± 0.09	0.86 ± 0.06	0.23 ± 0.02
5	3.68 ± 0.10	0.83 ± 0.04	0.38 ± 0.04
6	3.81 ± 0.08	0.78 ± 0.02	0.36 ± 0.03

*Results are presented as mean ± SD (n = 3). ND, not detected*.

**Table 5:** Organic acids production by the action of *Lactobacillus plantarum* L7 during the course of fermentation.

	**Organic acid production (mg g^−1^)**		
**Fermentation time (day)**	**Lactic acid**	**Acetic acid**	**Succinic acid**
0	ND	ND	ND
1	0.76 ± 0.04	0.16 ± 0.01	0.24 ± 0.02
2	1.64 ± 0.06	0.24 ± 0.01	0.28 ± 0.01
3	2.53 ± 0.07	0.29 ± 0.04	0.37 ± 0.03
4	4.95 ± 0.09	0.35 ± 0.03	0.19 ± 0.01
5	4.03 ± 0.10	0.36 ± 0.04	0.08 ± 0.01
6	3.69 ± 0.04	0.27 ± 0.03	ND

Ethanol production as a result of *L. plantarum* L7 metabolism was detected from the 2nd to the 6th day of fermentation, being the highest on day 5, followed by day 6 (Table 4). As the ethanol concentration was lower than 0.5% (w/v), the final product can be considered as a non-alcoholic beverage (Freire et al., 2017). Production of alcohol during the preparation of fermented rice-based beverages has been reported earlier (Ghosh et al., 2014; Ray et al., 2016).

### Lab growth performance during fermentation

**Figure 1:**
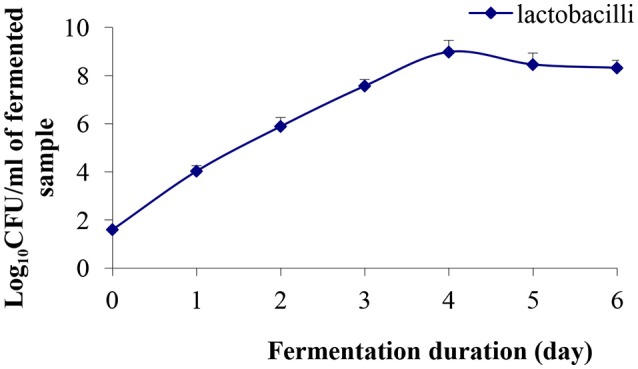
Evolution of lactobacilli population during the course of fermentation. Results are presented as mean ± SD (*n* = 3).

During fermentation, the growth of lactobacilli was enhanced exponentially upto day 4 (8.98 log CFU/mL), and thereafter, a slight decrease in the cell population was observed (Figure 1). At the end of the 6th day of fermentation, cell population was 8.32 log CFU /mL, which was above the minimum dose recommended for a probiotic product (6 log CFU /mL based on a daily dose of 100 mL) to confer a therapeutic effect (Sanders and Huis in't Veld, 1999). Recently, Ghosh et al. (2015b) reported that the LAB population reached 12.53 log CFU/mL in rice-based fermented beverage “haria” after 5 days of fermentation. Further, Rathore et al. (2012) reported *L. plantarum* population of 8.59 log CFU/mL when malt flour was fermented with this strain for 24 h. High viable counts are necessary to obtain the desired acid production and pH reduction, which affect the organoleptic properties of the product. Therefore, the observed production of lactic acid, minerals, and other metabolites, in this study, may be associated with higher lactobacilli population in the fermented product. LAB produced lactic acid and various metabolites, which inhibit the growth of intestinal pathogens (Jena et al., 2013) and have immunomodulatory activities.

### Amylase and glucoamylase activities

**Figure 2:**
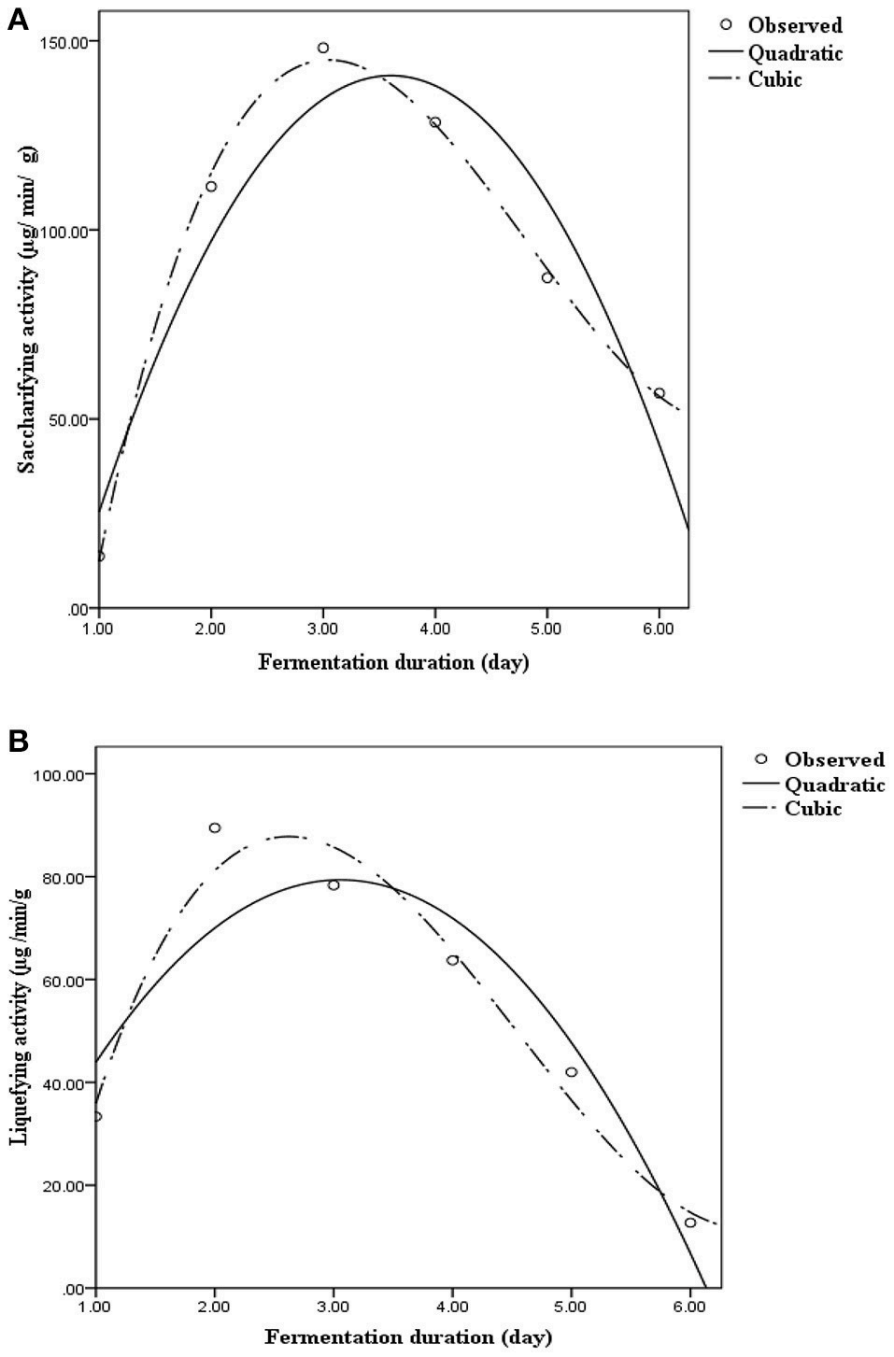
Saccharifying and liquefying activity on various days during rice fermentation. Results are presented through quadratic and cubic regression with respect to day. **(A)** Saccharifying activity = −80.133+122.605×Day−17.01×Day^2^ (R^2^ = 0.900); Saccharifying activity = −188.206+258.55×Day−62.04×Day^2^ + 4.24×Day3 (R^2^ = 0.997). **(B)** Liquefying activity = 1.03+51.25×Day−8.38×Day^2^ (R^2^ = 0.851); Liquefying activity = −66.09+135.68×Day−36.35×Day^2^ + 2.66×Day3 (R^2^ = 0.960).

Saccharifying (α-amylase) and liquefying (glucoamylase) activities during fermentation are presented in the form of quadratic/cubic equations in Figure 2. α-Amylase activity was found to be the highest on day 3 (148.13 μg/min g^−1^), and subsequently, decreased gradually, whereas glucoamylase activity peaked on day 2 (89.47 μg/min g^−1^), and thereafter, declined steadily. The curves (Figure 2) representing the variations in enzyme production as well as the corresponding *R*^2^-value exhibited that the cubic regression was best fitted to replicate actual variation. Lactobacilli are capable of producing various extracellular amylases that act on starch as a substrate (Petrova et al., 2013). Ghosh et al. (2015a,b) recently reported the involvement of lactobacilli in the production of α-amylase and glucoamylase during rice fermentation. These results suggested that probiotic microorganisms from traditional cereal-based fermented beverages could be exploited for use in the production of specific enzymes responsible for the fermentation of cereals.

### Phytase activity and mineral production

Phytate, which is present in rice, is considered harmful because of its strong propensity to complex with minerals such as Cu, Ca, Mg, Zn, and Fe, reducing their bioavailability. However, the breakdown of phytate can be encouraged by increasing the phytase activity (Baek et al., 2014). Baek et al. (2014) demonstrated that fermentation can eliminate phytate from rice through the production of phytase, and thus, increase mineral bioavailability. In the present study, phytase activity increased up to day 4 of fermentation (7.81 μg/min g^−1^), and then, decreased slowly (Table 6).

**Table 6:** Concentration of minerals during the course of rice fermentation.

**Fermentation time (day)**	**Phytase activity (μg min^−1^g^−1^)**	**Mineral contents (PPM)**				
		**Sodium**	**Calcium**	**Magnesium**	**Manganese**	**Ferrous**
0	*ND*	0.44 ± 0.41	0.78 ± 0.06	6.03 ± 0.13	0.03 ± 0.01	0.01 ± 0.005
1	0.79 ± 0.05	0.59 ± 0.03	0.81 ± 0.02	6.17 ± 0.1	0.09 ± 0.01	0.03 ± 0.005
2	1.11 ± 0.03	0.77 ± 0.02	1.02 ± 0.06	6.45 ± 0.06	0.16 ± 0.03	0.08 ± 0.01
3	3.64 ± 0.11	0.81 ± 0.06	1.34 ± 0.06	6.91 ± 0.06	0.30 ± 0.06	0.21 ± 0.02
4	7.81 ± 0.24	0.92 ± 0.04	1.16 ± 0.08	6.53 ± 0.08	0.09 ± 0.02	0.26 ± 0.03
5	6.39 ± 0.1	0.67 ± 0.02	0.92 ± 0.05	6.26 ± 0.09	0.016 ± 0.01	0.15 ± 0.01
6	4.31 ± 0.12	0.47 ± 0.04	0.74 ± 0.06	5.74 ± 0.07	0.01 ± 0.01	0.07 ± 0.01

*Values are shown as mean ± S.D (n = 3)*.

The body requires small quantities of mineral micronutrients and essential inorganic elements, such as those that are the main components of the bone and teeth, necessary for the regulation of metabolic processes and structural functions (Frazier, 2009). For instance, calcium provides skeletal rigidity and plays a vital role in many metabolic processes, sodium is important in maintaining water homeostasis and the functioning of nerves and muscles, iron is essential for the formation of blood cells, and magnesium is a cofactor of multiple enzymes involved in energy metabolism (Berdanier and Berdanier, 2015). In the present study, fermented rice was found to contain much higher levels of sodium (0.92 ppm on day 4 of fermentation), calcium (1.34 ppm on day 3), magnesium (6.91 ppm on day 3), manganese (0.30 ppm on day 3), and ferrous form of iron (0.26 ppm on day 4) than those in the unfermented rice (Table 6). The presence of minerals in traditional fermented rice beverages has also been described previously (Handique et al., 2017). These results suggested that degradation of phytate during fermentation by *L. plantarum* L7 led to the availability of these minerals.

### Analysis of sugars in fermented rice

**Figure 3:**
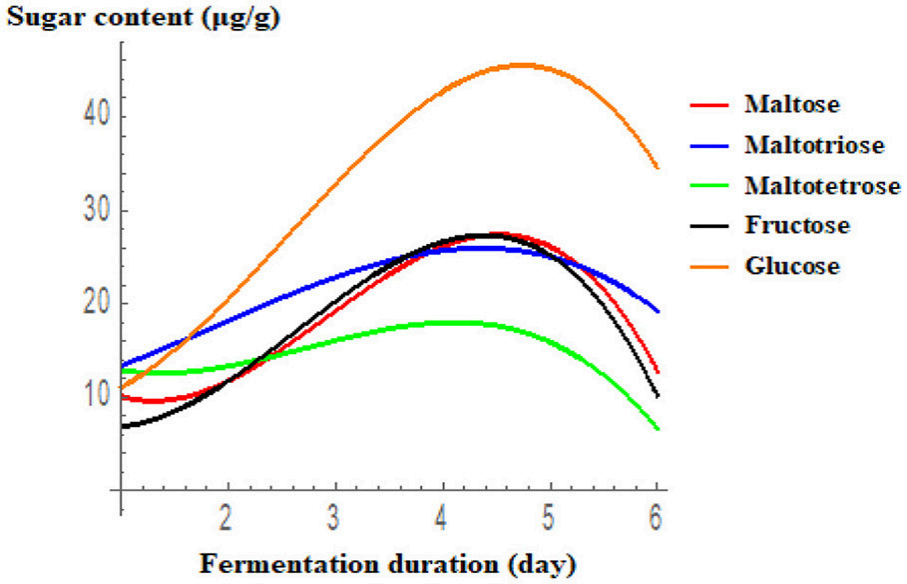
Accumulation of different sugars during the rice fermentation with respect to day. Results are presented using cubic regression. Maltose = 20.61−18.681D+9.257D^2^−1.060D^3^ (R^2^ = 0.956); Maltotriose = 10.133+1.983D+1.585 D^2^−0.277D^3^ (R^2^ = 0.978); Tetrose = 8.009−8.871D+4.326 D^2^−0.527D^3^ (R^2^ = 0.937); Fructose = 11.322−10.99D+7.518 D^2^−.953D^3^ (R^2^ = 0.98); Glucose = 9.767−4.462D+6.665 D^2^−0.872 D^3^ (R^2^ = 0.986).

The results of sugar analysis by HPLC are presented in Figure 3. Glucose, maltotriose, maltotetrose, and fructose concentrations peaked on the 4th or 5th day of fermentation, and then decreased gradually. Ghosh et al. (2015a,b) recently reported the presence of various sugars in the traditional fermented rice beverage, haria, but their quantities were slightly lower when rice was fermented with a single LAB isolated from this drink. Similarly, glucose, fructose, sucrose, and maltose were identified in the fermented food, calugi (Miguel et al., 2012). In the present study, activity of the starch hydrolytic enzymes, glucoamylase and amylase, was the highest on the 2nd and 3rd day, respectively. According to Ghosh et al. (2015b), starch is first converted to limit dextrin by α-amylase, followed by the specific production of glucose from this fragmented polysaccharide by glucoamylase. Thus, the synergistic activity of amylase and glucoamylase breaks down starch into malto-oligomers and glucose (Almeida et al., 2007). Consumption of malto-oligomers, such as maltotriose and maltotetrose, is beneficial for human health. Production of such oligosaccharides due to the activity of *Lactobacillus* enzymes in rice-based fermented beverages has also been documented in other studies (Ghosh et al., 2015b; Puerari et al., 2015).

### Determination of antioxidant activity

In this study, ABTS^+·^ and DPPH scavenging activities increased gradually up to the 4th and 5th days of fermentation, respectively, and then declined (Table 7). RSA was significantly higher in fermented rice (i.e., days 2–6) than in unfermented rice. The observed changes in RSA may be related to the higher concentrations of phenolic compounds and flavonoids found in the fermented rice (Supplementary Figure 1). Phenolics, including isoflavones, widely distributed in plants, are recognized as the most abundant antioxidants in the human diet (Xiao et al., 2015). In a recent study on the traditional fermented rice-based beverage, haria, Ghosh et al. (2015a) demonstrated a much higher level of antioxidant activity (82.54%) against DPPH free radicals. In a previous investigation, rice fermented using a starter culture of *L*. *fermentum* KKL1 exhibited DPPH RSA (52.34%) (Ghosh et al., 2015a) which is consistent with the result obtained in the current study. Further, soy whey fermented with *L. plantarum* B1–6 exhibited good antioxidant activity (Xiao et al., 2015). The elevated antioxidant activity suggested that this fermented product may have health benefits.

**Table 7:** Radical scavenging activities of fermented rice as measured by DPPH and ABTS assays.

**Fermentation days**	**DPPH scavenging activities (%)**	**ABTS scavenging activities (%)**
0	7.36 ± 0.35^a^	9.3 ± 0.47^a^
1	13.16 ± 0.7^b^	11.4 ± 0.31^a^
2	22.43 ± 1.04^c^	17.18 ± 0.43^b^
3	36.26 ± 1.32^d^	28.62 ± 0.61^c^
4	51.9 ± 1.2^e^	43.82 ± 0.68^d^
5	62.56 ± 0.7^f^	42.1 ± 0.32^d^
6	47.46 ± 1.03^e^	29.84 ± 0.41^c^

*Results are presented as mean ± SD (n = 3). Values in the same column with different superscripts letters are significantly different (P < 0.05)*.

### GC-MS analysis

The volatile compounds in fermented rice sample were analyzed by GC-MS (Table 8; Supplementary Figure 2). In total, 23 compounds including various esters, alcohols, acids, and other compounds were detected. The relative peaks represent percentages (approximately) of the compounds identified in fermented rice sample. The total relative peak areas are as follows: alcohols (20.3%), esters (61.82%), acids (13.87%), and aldehydes (4.01%). The total peak areas of volatile esters were higher than those for acids and alcohols. The esters of various acids were the largest group of compounds identified in fermented rice. The majority of esters were formed by esterification during fermentation. Formation of various esters during rice fermentation has been reported previously (Kim et al., 2010, 2013). Succinic acid and octanoic acid ethyl esters were the most abundant in the rice sample with peak areas 28.11 and 23.58%, respectively.

**Table 8:** Compound identified fermented rice by GC-MS analysis.

**No**.	**Retention time**	**Compounds**	**Peak area (%)**
1	3.491	Ethanol	3.48 ± 0.28
2	6.092	Butanoic acid	0.25 ± 0.02
3	10.83	Propanoic acid	0.32 ± 0.03
4	11.27	Diethylene glycol, DI-TMS	0.26 ± 0.09
5	11.48	Pentanoic acid	0.55 ± 0.05
6	11.76	Isoamyl alcohol	8.17 ± 0.5
7	11.82	Heptanoic acid, ethyl ester	3.77 ± 0.29
8	13.20	2-Propanol	6.28 ± 0.59
9	13.55	Glycerol	0.55 ± 0.045
10	15.26	Propanedioic acid	12.01 ± 1.2
11	15.45	Lactic acid ethyl ester	0.48 ± 0.048
12	16.63	Glutamic acid ethyl ester	0.25 ± 0.18
13	17.83	Octanoic acid ethyl ester	23.58 ± 2.0
14	19.37	Butanol	1.56 ± 0.1
15	19.47	2-Pyrrolidone carboxylic acid ethyl ester	0.64 ± 0.06
16	19.64	Benzaldehyde	4.01 ± 0.35
17	19.83	Nonanoic acid ethyl ester	0.27 ± 0.025
18	21.17	Hexadecanoic acid	0.26 ± 0.028
19	22.49	Decanoic acid ethyl ester	3.49 ± 0.28
20	21.55	Octanoic acid, Trimethylsilyl ester	0.64 ± 0.062
21	22.77	9,12-Octadecadienoic acid	0.48 ± 0.045
22	23.49	Hexadecanoic acid	0.59 ± 0.056
23	26.19	Succinic acid diethyl ester	28.11 ± 2.8

*Peak area are presented as mean ± SD of two independent experiments*.

Isoamyl alcohol and ethanol were the most abundant with peak areas 8.17 and 3.48%, respectively. Isoamyl alcohol is an important component in high quality alcoholic beverages, strongly impacting the aroma and taste by contributing a sweet, banana-like aroma (Yuda, 1976). Isoamyl alcohol is important for aromatic profile of several beverages, such as wines and beers (Riu-Aumatell et al., 2014).

Propanedioic acid was the most abundant acid in the sample with peak area 12.01%. Butanoic acid, another volatile compound, was produced during fermentation and was present in very less quantity (0.25% peak area). This compound has an unpleasant smell and acrid taste, being responsible for rancid butter-like aromas, and therefore, it is undesirable (Freire et al., 2017). Further, hexadecanoic acid was detected at the retention time 21.17 min (0.26% peak area), gives rice beers a barely detectable, slightly waxy, creamy, and sweet taste (Kim et al., 2010).

Benzaldehyde was the only aldehyde identified in the fermented sample. The peak area was 4.01%. Benzaldehyde formation is also reported in yakju prepared from yeast strains (Kim et al., 2010). Presence of various alcohols, acids, and aldehydes in fermented rice beverages has been detected by GC-MS previously (Kim et al., 2010, 2013; Freire et al., 2017). Further, presence of organic acids in fermented rice beverages detected by HPLC (described in earlier section) and GC-MS, contributes to the refreshing flavor, unique aroma, and texture, beside controlling the growth of food spoilage microorganisms (Duarte et al., 2010).

## Conclusions

In this study, 9 LAB isolates from traditional rice-based fermented beverage “bhaati jaanr” were evaluated for potential probiotic properties. Those isolates were found to have varying pH and bile salt tolerance, cell surface hydrophobicity, auto-aggregation, antibiotic susceptibility, and antibacterial activities. Our results indicated that one of the isolates, identified as *L. plantarum* L7, possessed desirable *in vitro* probiotic properties. The role of this strain as a starter culture in the preparation of rice-based fermented beverage was evaluated. This strain increased the functional component (nutrients and minerals) levels of the fermented beverage. Interestingly, this bacterium enhanced the mineral bioavailability via heightened phytase activity. Rapid acidification and the generation of organic acids by *L. plantarum* L7 improve the safety of the resulting product. Fermentation with this strain improved the digestibility of the final product by reducing starches and increased its antioxidant potential. Currently, beneficial effects of *L. plantarum* L7 are under investigation *in vitro* and *in vivo* to establish its probiotic characteristic.

## Author contributions

SG and VS designed the study. SG conducted the study and wrote the manuscript. SSS helped in GC-MS analysis and reviewed the manuscript. SS contributed to the interpretation of data. VS and SP supervised and critically reviewed the manuscript.

### Conflict of interest statement

The authors declare that the research was conducted in the absence of any commercial or financial relationships that could be construed as a potential conflict of interest.
