# Supplementary material for: Use of a Potential Probiotic, Lactobacillus plantarum L7, for the Preparation of a Rice-Based Fermented Beverage
Source: Front Microbiol. 2018 Mar 14;9:473. doi: 10.3389/fmicb.2018.00473 (PMC5861207; doi:10.3389/fmicb.2018.00473)

SUPPLEMENTARY FIGURES

Supplementary Figure 1.

Phenolics and flavonoid concentrations during fermentation of rice. Results are presented through power regression equation.

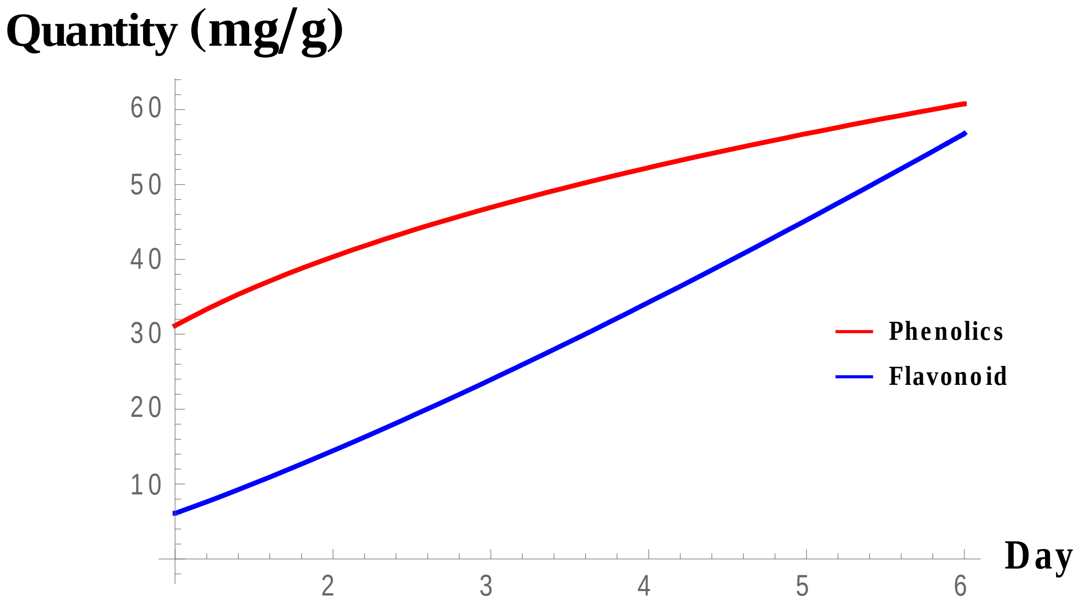

Phenolics = $31.154(0.373)^{Day}$ ($R^2 = 0.962$)

Flavonoid = $6.090(1.246)^{Day}$ ($R^2 = 0.911$)

Supplementary Figure 2. GC-MS chromatogram of fermented rice-rice sample

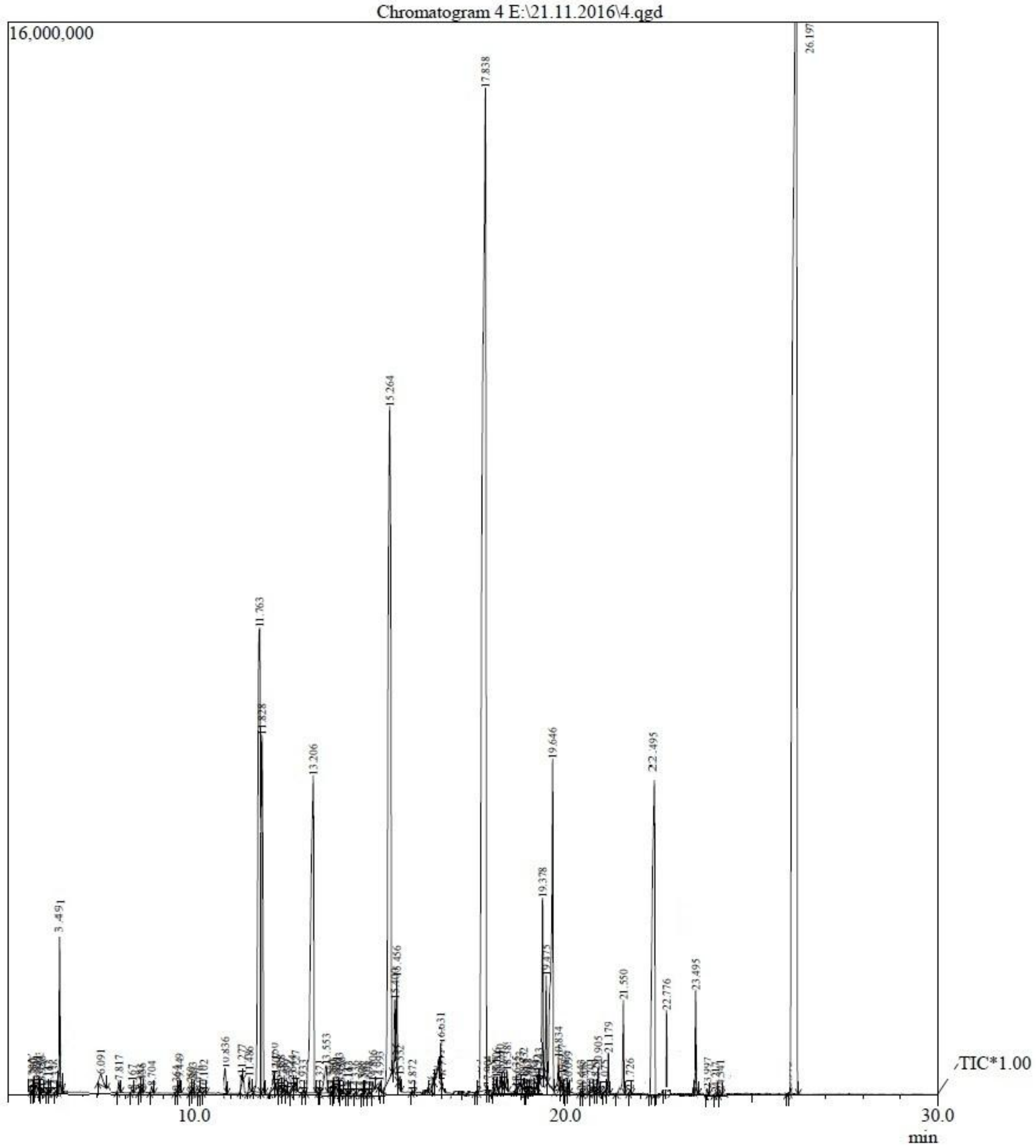

Supplement: Supplementary file 1 [file Image1.PDF]
